# Evaluating Andrographolide as a Potent Inhibitor of NS3-4A Protease and Its Drug-Resistant Mutants Using *In Silico* Approaches

**DOI:** 10.1155/2015/972067

**Published:** 2015-10-26

**Authors:** Vivek Chandramohan, Anubhav Kaphle, Mamatha Chekuri, Sindhu Gangarudraiah, Gowrishankar Bychapur Siddaiah

**Affiliations:** Department of Biotechnology, Siddaganga Institute of Technology, Tumkur, Karnataka 572 103, India

## Abstract

Current combination therapy of PEG-INF and ribavirin against the Hepatitis C Virus (HCV) genotype-1 infections is ineffective in maintaining sustained viral response in 50% of the infection cases. New compounds in the form of protease inhibitors can complement the combination therapy. Asunaprevir is new to the drug regiment as the NS3-4A protease inhibitor, but it is susceptible to two mutations, namely, R155K and D168A in the protein. Thus, in our study, we sought to evaluate Andrographolide, a labdane-diterpenoid from the *Andrographis paniculata* plant as an effective compound for inhibiting the NS3-4A protease as well as its concomitant drug-resistant mutants by using molecular docking and dynamic simulations. Our study shows that Andrographolide has best docking scores of −15.0862, −15.2322, and −13.9072 compared to those of Asunaprevir −3.7159, −2.6431, and −5.4149 with wild-type R155K and D168A mutants, respectively. Also, as shown in the MD simulations, the compound was good in binding the target proteins and maintains strong bonds causing very less to negligible perturbation in the protein backbone structures. Our results validate the susceptibility of Asunaprevir to protein variants as seen from our docking studies and trajectory period analysis. Therefore, from our study, we hope to add one more option in the drug regiment to tackle drug resistance in HCV infections.

## 1. Introduction

More than 25 years after the discovery, Hepatitis C Virus (HCV) is still considered a major global threat to human health. The viral infection is spread over 130–170 million people worldwide [1] and a significant number of people, around 350,000 to 500,000, die each year because of Hepatitis C related liver diseases according to the WHO [2]. Combination of pegylated interferon-*α* (PEG-INF) with ribavirin has been used as a major treatment for the infection [3, 4]. However, 50% of the HCV genotype-1 infected individuals do not show a sustained virological response (SVR) for the combination, reasons of which have been recently explored by Padmanabhan et al., using systems biology approaches [5]. Several factors have been identified that correlate with these nonresponsive observations, some of which are found to be the genomic differences between individuals, viral genotype, and single-nucleotide polymorphisms (SNPs) in the interferon-*λ* locus [6, 7]. New drug compounds in the form of protease and polymerase inhibitors are currently in the development as the direct-acting antivirals (DAAs). Studies have shown that, together with the combined therapy of PEG-INF with ribavirin, these antiviral compounds have shown to increase SVR from less than 50% to around 70% in HCV genotype-1 patients [8]. However, the potential of these DAAs has been obscured by high mutation rates and genomic heterogeneity in the virus [9]. Introduction of frequent mutations in the viral genome due to the infidel nature of viral replicase adds plights to drug researchers looking for new antiviral.

Viral proteases are vital for infection and proliferation and hence they can be considered as potential targets for DAAs to intervene viral cycle. In our work, we have selected NS3-4A protease which is responsible for cleaving single precursor polypeptide, together with NS2-NS3 and NS3 proteinases, of length 3010-3011 aa translated from the long reading frame to yield active proteins [10–12]. Many proteases inhibitors like telaprevir or boceprevir have been approved by the FDA as the potent inhibitors of the protease; however, the mutations in the protein have led to rapid drug inefficacy [13, 14]. Asunaprevir is yet another effective protease inhibitor being developed by Bristol-Myers Squibb and is in its 3rd clinical trial phase. However, the binding capacity of Asunaprevir has been limited by two mutations in the protein structure, namely, R155K and D168A [15]. Crystal structure of the proteases is available publicly on the Protein Data Bank (PDB) website and structure-based drug design approach can be applied to screen plethora of new DAAs that can have maximum binding efficiency against any concomitant mutations in the proteins. Plants are considered as great source of medicinal compounds, and they can be explored to drive drug discovery process fast and smoothly with minimum budget concern [16].* Andrographis paniculata *Nees is an herbaceous plant in the family Acanthaceae. It has a broad range of pharmacological effects which also include antiviral activity [17–19]. The plant extract contains various phytochemicals majority of which are diterpenoids and flavonoids. Andrographolide, a labdane-diterpenoid, is a major bioactive compound from the plant extract [20].

Thus, our work is directed towards exploring the binding potential of Andrographolide from the plant against the mutations in the protein by molecular docking, dynamics, and comparing its effects with Asunaprevir computationally.

## 2. Material and Methods

### 2.1. Protein Preparation

3D structure of wild-type NS3 protease was retrieved from the Protein Data Bank (PDB) using query ID 4NWL [21]. Cocrystallized ligand, water molecules, and zinc ions were removed from the target structure to obtain clean protein [22]. The protein mutants were prepared by replacing the native residues in the protein with the mutant residues (R155K and D168A) [23] using DS 3.5 “build mutants” option. The structures thus obtained were optimized classically using CHARMm force field implemented in the DS 3.5, minimized with conjugate gradient energy minimization protocol followed by convergence energy minimization (0.001 kcal/mole), that readied the structures for docking and simulations [24]. Active site residues (*Q41, F43, H57, G58, D81, R109, K136, G137, S138, S139, G140, G141, F154, R155, A156, A157, D168, M485, V524, Q526, and H528*) [25] were selected for both the wild-type protein and mutant structures for molecular docking studies.

### 2.2. Ligand Preparation

The investigated compounds Andrographolide and Asunaprevir were drawn using Marvin sketch [26]. Ligand optimization was carried out using Chemistry at Harvard Molecular Mechanics (CHARMm) and macro molecular force field (MMF) followed by energy minimization protocol [27]. Several ligand conformations were generated based on bond energy, CHARM energy, dihedral energy, electrostatic energy, initial potential energy, and initial RMS gradient values. The drug likeliness was evaluated using the Lipinski rule of 5 via Lipinski drug filter protocol [28]. The studies on the ADME of aqueous solubility, blood brain barrier level, hepatotoxicity, plasma protein binding levels, and CYP2D6 were carried out [29]. Toxicity profile of the ligand molecules was predicted by using TOPKAT which applies a range of robust, cross validated, and Quantitative Structure-Toxicity Relationship (QSTR) models for assessing specific toxicological endpoints. The toxicity profile also included NTP carcinogenicity, mutagenicity, and developmental toxicity and skin irritation assessment [30]. The studies were performed using Discovery studio 3.5 (Accelrys).

### 2.3. Molecular Docking and Dynamics

For molecular docking studies, a flexible docking approach was employed using the LeadIT [31] software in which wild NS3 protease and mutants R155K and D168A were considered as receptor proteins. The docking results for receptor-ligand complex comprised intermolecular interaction energies, namely, hydrogen bonding and hydrophobic and electrostatic interaction. Receptor-ligand complex with least binding energy was used to infer the best binding compound. Molecular dynamics (MD) simulations for both proteins and ligands were performed in a flexible manner that allowed binding site to be relaxed around the ligand and directly estimate the effect of explicit water molecules. MD-based computational techniques are available for estimating the binding free energy which includes thermodynamic integration (TI), free energy perturbation (FEP), linear interaction energy (LIE), and molecular mechanics/Poisson-Boltzmann and surface area (MM/PB-SA) methods. Three best receptor-ligand complexes were subjected to molecular dynamics studies based on steepest decent minimization protocol. For dynamics study, the following parameters, heating steps and time steps set as 2000 and 0.001, respectively, equilibration steps and time steps set as 1000 and 0.001, respectively, for the overall production period of 20 ns with time steps as 0.001 and temperature factor of 300 K, were considered. The best conformations were selected based on the least potential energy value [32].

## 3. Result and Discussions

### 3.1. Protein Preparation

**Figure 1:**
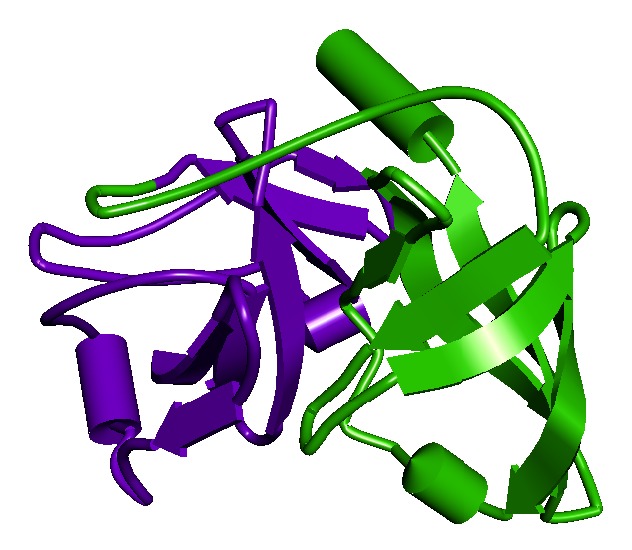
The figure depicts the structure of NS3 protease with schematic model. Violet color shows the N-terminal serine protease domain region in the protein, and green color shows the C-terminal domain region that is a member of the DExH/D-box helicase superfamily II with NTPase activity.

**Figure 2:**
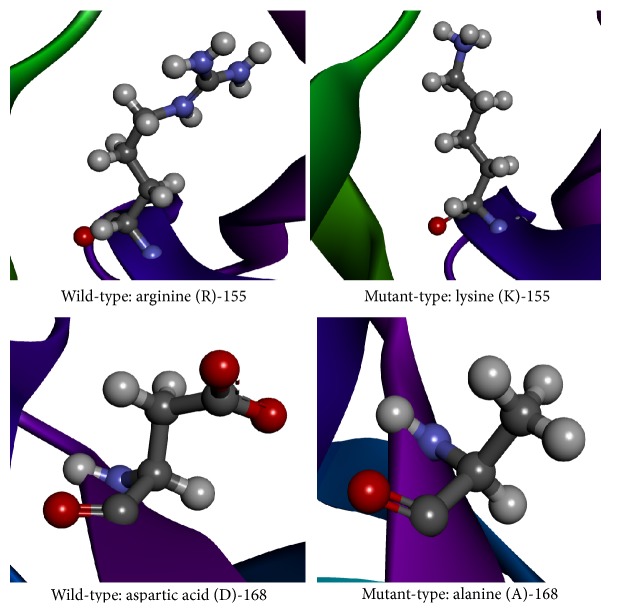
The figure depicts the native and mutated residues (shown in balls-and-stick model) of the variants R155K and D168A in the structure of NS3 protease (color rainbow). As can be inferred, the change in the residues introduced larger amino acid groups for R155K that will decrease the binding efficiency of drug molecules due to more steric hindrances. Also, the introduction of nonpolar groups for D168A transition will contribute to only weaker molecular interactions and thus reduced binding.

**Figure 3:**
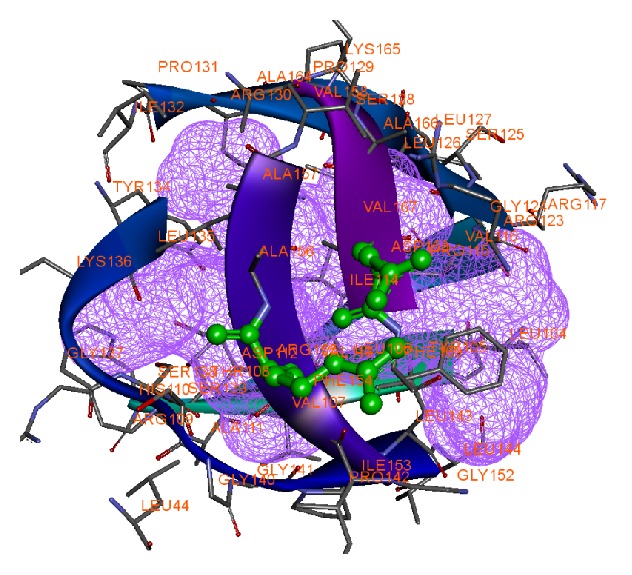
Active site of target protein with mutant structure shown in ball-and-stick model of variants R155K and D168A (violet wire mesh indicates binding cavity in the active site).

The obtained protein structure has a single-chain construct of protease domain of Hepatitis C Virus genotype-1a, with a covalently linked cofactor 4A at the N-terminal [21]. The protease belongs to the hydrolase class in the Enzyme Commission (EC) classification with EC number 3.4.21.98. It is a bifunctional enzyme that has two domains depicted in Figure 1, namely, the N-terminal serine protease domain that locates between −7 and 87 aa and C-terminal domain that is a member of the DExH/D-box helicase superfamily II with NTPase nucleic acid binding and helicase unwinding activities, located between 88 and 182 aa. The “build mutant” option in the DS generated single optimized structures for the mutations R155K and D168A with Discrete Optimized Potential Energy scores [33] (DOPE scores) of −19975.94 and −20031.18, respectively. The change in the amino acids backbone has been compared by keeping the structures side-by-side as shown in Figure 2. The figure clearly shows the difference in the backbone structure and it can be inferred that the change may cause an increase in the steric hindrance for binding of drug molecules. The active site residues have been taken from the PDB records of the structure.  Figure 3 shows the structural conformation of the residues in and around the active site. It clearly shows the cavity in the structure where our ligand molecules are expected to fit.

### 3.2. Ligand Preparation

Andrographolide is a labdane-diterpenoid compound which is known for its wide range of pharmacological potential. It has been shown to have antiviral, antimalarial activities. Thus, we have considered it as a potent compound for tackling drug resistance in the HCV infection and compared its potency against the mutation-sensitive Asunaprevir. The two-dimensional structure and molecular properties of investigated compounds were tabulated in Table 1. The possible 3D conformations generated for Asunaprevir were 1 and for Andrographolide were 16. Out of the generated conformations, the lowest potential energy was selected for further studies. Conformity with ADME and TOPKAT prediction is shown in Tables 2 and 3. Both the compounds are predicted to be safe and show very less toxicity. Asunaprevir has been predicted to be slightly hepatotoxic; however, it should be noted that the predictions are defined based on certain established algorithms and may not be sometimes reliable in the real setup, which is plausible as Asunaprevir has already passed the initial phases of clinical trials (i.e., I and II). The mutagenicity level of both the compounds is also predicted to be low and thus both are predictively nontoxic for any systemic administration.

### 3.3. Molecular Docking and Dynamics

**Figure 4:**
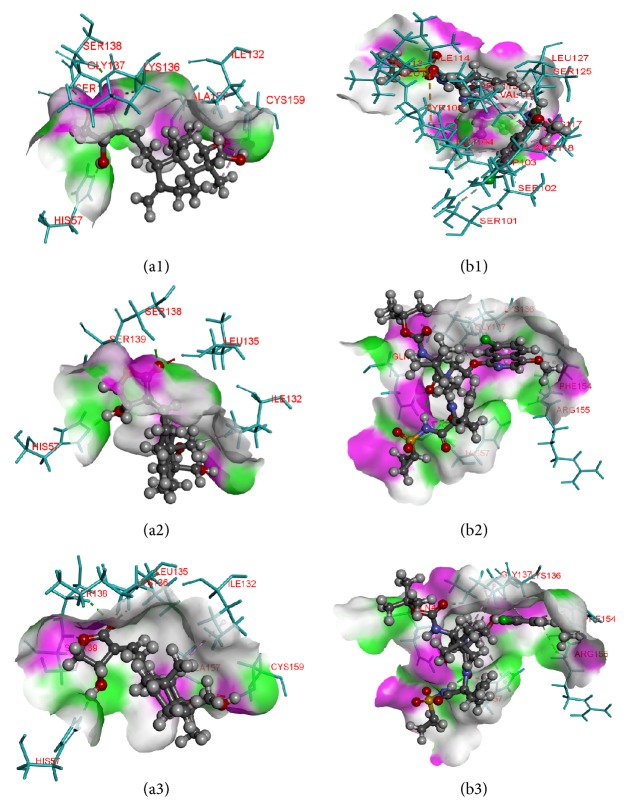
Binding poses and atomic interactions between ligands and receptors. (a1, a2, a3) series depicts Andrographolide interactions with the wild-type mutant R155K, and mutant D168A, respectively. (b1, b2, b3) series depicts the same with Asunaprevir and mutants in the same order above (see text for interacting residues). Note: ligands shown in ball-and-stick pattern and interacting residues shown in stick pattern, protein surface. Pink: donor, green: acceptor.

Molecular docking is an efficient technique to predict the preliminary binding modes of ligand with the protein of solved three-dimensional structure. Studies on binding poses are essential to elucidate key interactions between the small molecules and receptors and they provide helpful data for designing effective inhibitors. In our study, flexible docking method was used, using Biosolve LeadIT to dock compounds into active site of the protein structures. The rationale of using flexible docking is to give compounds enough flexibility to attain all the possible 3D space conformation and not to restrict only certain rigid structures. Docking results showed that Andrographolide occupies binding region of the native protein as well as its structural variants effectively with higher docking score than Asunaprevir. The detailed overview of the binding scores and interacting residues are shown in Table 4. Also the docking poses of ligand-receptor interaction are depicted in Figure 4. Lead-IT docking score correlates with the free binding energy. Andrographolide binds the native protein with a Lead-IT score of −15.0862 and interacts with three amino acid residues, namely, SER138, SER139, and HIS57, via hydrogen bonding. In the R155K mutated structure, the compound forms 6 hydrogen bonds with residues SER138, SER139, ALA157, HIS57, LYS136, and GLY137 with docking score of −15.2322. Similarly, the compound has docking score of −13.9072 with the D168A mutated structure and again interacts through 6 hydrogen bonds with amino acid residues, namely, SER138, SER139, ALA157, HIS57, LYS136, and SER139. In all of the protein structures, Asunaprevir has low binding scores, lowest with the R155K mutation with the score of just −2.6431. It was expected because of the high susceptibility towards the mutation as described in various literatures; the reason is the fact that Asunaprevir makes contacts with R155 residue outside the substrate envelop which is thus stabilized by the D168 residue, and thus any mutation in either of residues will disrupt the interactions between Asunaprevir and the enzyme [15]. Our results thus show that Andrographolide has better binding ability with the protein structures than Asunaprevir.

**Figure 5:**
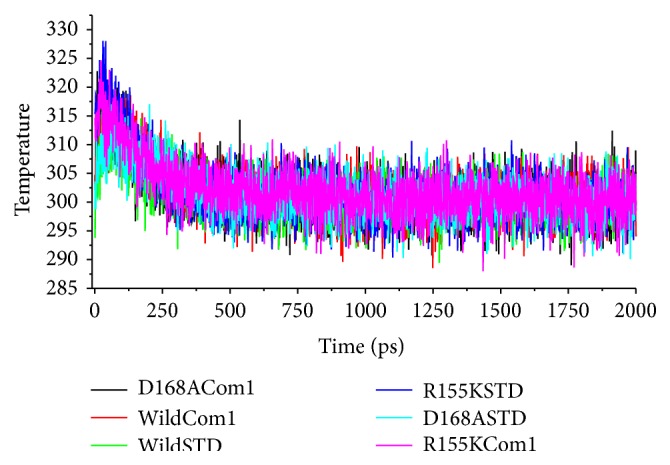
Temperature equilibration of the ligand-protein systems. As can be seen from the plots, the systems for all the ligand-protein complexes readily attained the temperature set at 300 K and maintained it throughout the simulation period. (Note: Asunaprevir is mentioned as STD and Andrographolide as Com 1.)

**Figure 6:**
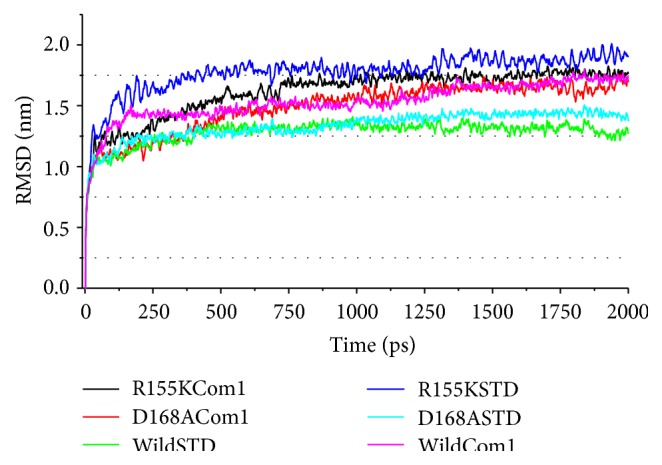
Protein backbone RMSD calculation plots for ligand bound complexes. Asunaprevir perturbs backbone of the protein mutant R155K (curve in blue) more than Andrographolide (curve in black). Surprisingly, Andrographolide seems to disturb protein structure for the mutant D168A more than Asunaprevir (check cyan curve for Asunaprevir and red for Andrographolide). (Note: Asunaprevir is mentioned as STD and Andrographolide as Com 1.)

**Figure 7:**
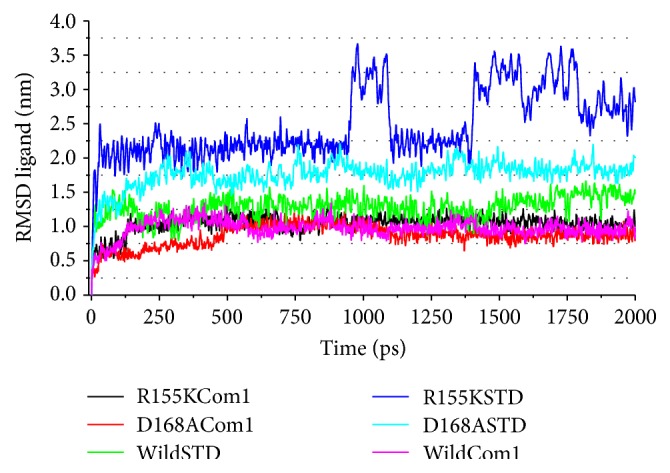
Ligand RMSD calculation plots for ligand bound complexes. The curves colored in blue and cyan show the instability of Asunaprevir inside the binding cavity of R155K and D168A mutants, respectively, while good stability is seen in wild type as shown by the green curve. Andrographolide is relatively very stable with very less deviation in the RMSD values for all the complexes (curves colored in black for R155K, red for D168A, and pink for wild-type structures, resp.). (Note: Asunaprevir is mentioned as STD and Andrographolide as Com 1.)

To compare the structural behavior and flexibility of the wild-type and mutant proteins, both the lead compounds were incorporated in Discovery studio MD simulations run and the studies were performed for 20ns for each complex with all the parameters as mentioned in Materials and Method. The dynamic simulation runs create a system that tries to mimic physiological environment to check if the ligand is really stable within the cavity of target protein, maintain bonds, and be able to inhibit the activity for a certain period of time which will result in therapeutic actions. As can be seen, the ligand-protein systems readily attained the given temperature of 300 K and stayed approximately around it throughout the run (Figure 5). Root mean square deviations (RMSD) [34] of the wild-type and the mutants were calculated against their initial structure in the protein-ligand complexes and graphs were generated to compare the flexibility once the ligand is bound to the structure. Over the simulation period, the backbone of the proteins remained fairly stable, as the graph shows in Figure 6. The binding of Asunaprevir did not disturb protein backbone stability in D168A and wild protein structures. However, in the mutant structure R155K, the binding caused a considerable perturbation in the backbone with RMSD value eventually deviating by 0.5 nm in the end. Andrographolide did disturb the backbone when compared to Asunaprevir in both wild-type and D168A mutant. However, in case of R155K mutant structure, binding of Andrographolide did not disturb the backbone much as compared to Asunaprevir implying that Andrographolide binds to the mutant stably. This may be because of the small molecular size of Andrographolide that gives it enough freedom in space, whereas Asunaprevir, given its size and flanging chemical moieties, would not have more freedom, and within short simulation period the steric hindrances between the atoms of Asunaprevir and protein start making the system instable. To ensure the binding stability of the drug candidates in the active site of proteins, ligand positional RMSD of each lead molecule were generated and plotted. As can be seen from Figure 7, Asunaprevir showed more fluctuations in noticeable size of 2.0–3.5 nm with the R155K mutant. Also, it was not stably binding with D168A mutant when compared to our ligand molecule; however, the binding stability with the wild type was stable with very low deviations. Andrographolide showed stability in binding to all of the protein structures.

## 4. Conclusions

Most direct-acting antivirals are directed towards inhibiting proteases and polymerases. NS3-4A serine protease of the HCV is one of the most interesting targets and has a key role in HCV infection and proliferation. A good number of antivirals to inhibit this protease are already in the clinical trial phases, among which Asunaprevir stands in the first line of competitive inhibitors targeting HCV serine protease NS3-4A. However, the resulting side effects and sensitivity of the drug towards the HCV mutants R155K and D168A limit its potential. In this study, we compared the interaction efficiency of Asunaprevir and diterpenoids Andrographolide with the wild-type HCV protease and its mutants. The molecular docking studies using LeadIT revealed that the Asunaprevir binds with docking scores of −3.7159, −2.6431, and −5.4149, and Andrographolide binds with docking scores of −15.0862, −15.2322, and −13.9072, to the wild-type R155K and D168A structures, respectively. It infers that Andrographolide can interact strongly with the protein's active site residues both in the wild type and in mutants with least energy compared to Asunaprevir. The stability of the ligand-protein complexes was evaluated from the molecular dynamic simulations tool in the DS 3.5. Using calculated backbone RMSD data, it was found that Asunaprevir maintains protein stability in both the wild-type and D168A structures and, however, disturbs R155k backbone. Andrographolide did perturb the backbone in both the wild and mutant D168A structures but does not cause much disturbance in the mutant structure R155K when compared to Asunaprevir. We used ligand RMSD calculation data to infer about the binding stability of ligands with the structures. Asunaprevir showed more fluctuations in R155K complex than in others. Andrographolide was binding stably in all the structure types inferring the interactions are strong. Therefore, our study reports that Andrographolide can act as a promising option to target and inhibit NS3-4A along with its drug resistive mutants.

## Figures and Tables

**Table 1:** Structure of ligands with their molecular properties.

SN	Compound name	Properties	2D images
1	Andrographolide	Compound ID: 5318517 Molecular weight: 350.4492 (g/mol) Molecular formula: C_20_H_30_O_5_ XLogP3: 2.2 Hydrogen bond donor count: 3 Hydrogen bond acceptor count: 5	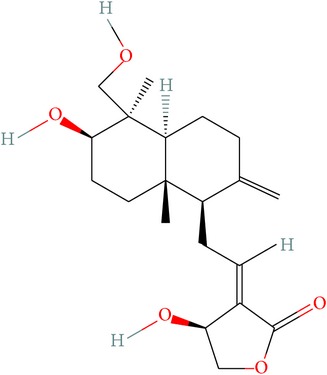

2	Asunaprevir	Compound ID: 16076883 Molecular weight: 748.28584 (g/mol) Molecular formula: C_35_H_46_ClN_5_O_9_S XLogP3: 4.9 Hydrogen bond donor count: 3 Hydrogen bond acceptor count: 10	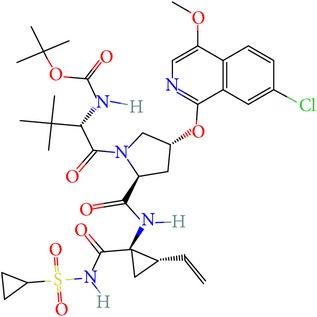

**Table 2:** Comparison of the ADME values of ligands.

Name	Solubility level	Blood brain barrier level	Extension CYP2D6	Extension hepatotoxic	Extension PPB
Andrographolide	2	3	−1.54262	−10.8965	16.7621
Asunaprevir	2	4	−9.92277	3.66033	24.0848

Note: solubility: 0–2: highly soluble, BBB: 1: high penetration, 2: medium penetration, and 3: low penetration, CYP2D6: −ve: noninhibitors and +ve: inhibition. HEPATOX: <1: nontoxic, PPB: the greater the value, the greater the binding capacity.

**Table 3:** Comparison of the predicted TOPKAT values of ligands.

Name	NTP carcinogenicity call (male mouse) (v3.2) TOPKAT	NTP carcinogenicity call (female mouse) (v3.2) TOPKAT	Developmental toxicity potential (DTP) (v3.1) TOPKAT	Skin irritation (v6.1) TOPKAT	Ames mutagenicity (v3.1) TOPKAT
Andrographolide	0.00	0.00	0.00	1.00	1.00
Asunaprevir	0.00	1.00	0.00	1.00	1.00

Note: 0: negative result, 1: positive result.

**Table 4:** Ligand-protein interaction with docking scores.

Protein	Compound name	Lead-IT (docking)					
		Lead-IT score	H-bond	Amino acid	Amino acid atom	Ligand atom	H-bond length (Å)
Wild-type HCV protease	Andrographolide	−15.0862	3	SER138	HN	O5	1.99421
				SER139	HN	O3	2.17439
				HIS57	NE2	H55	1.719
	Asunaprevir	−3.7159	5	GLY41	HE21	O8	1.6653
				HIS57	HD2	O6	2.12614
				GLY58	HA1	O6	3.07324
				GLY137	HA1	O7	2.76771
				ARG155	O	H92	2.73609

R155K	Andrographolide	−15.2322	6	SER138	HN	O5	2.44552
				SER139	HN	O3	2.29348
				ALA157	O	H53	1.79921
				HIS57	NE2	H55	2.17054
				LYS136	HA	O5	2.5112
				GLY137	HA1	O3	3.09817
	Asunaprevir	−2.6431	6	TYR105	HH	O10	2.87824
				LEU106	HN	O3	1.83884
				SER125	HN	O11	2.00467
				LEU104	O	H95	1.59587
				SER101	HB2	CL1	2.96175
				SER125	O	H80	2.7483

D168A	Andrographolide	−13.9072	6	SER138	HN	O5	2.27611
				SER139	HN	O3	2.20946
				ALA157	O	H53	2.59575
				HIS57	NE2	H55	1.6014
				LYS136	HA	O5	2.20148
				SER139	HB2	O3	3.02411
	Asunaprevir	−5.4149	8	GLN41	HE21	O8	1.65812
				HYS57	O	H97	1.55407
				HYS57	HD2	O6	2.06801
				LYS136	HE21	O10	2.77644
				GLY137	HA1	O7	2.87646
				ARG155	O	H80	3.0789
				ARG155	O	H92	2.76844
				GLY137	HN	06	2.64645
